# Identification of IRF8 as an immune infiltration‐related biomarker in hepatocellular carcinoma by bioinformatics analysis

**DOI:** 10.1002/mco2.149

**Published:** 2022-07-10

**Authors:** Renyu Zhang, Yixiao Guo, Zekun Liu, Lingmin Kong, Can Li, Lin He, Cong Zhang, Zhinan Chen, Huijie Bian, Ding Wei

**Affiliations:** ^1^ Department of Cell Biology National Translational Science Center for Molecular Medicine Fourth Military Medical University Xi'an China

Dear Editor,

Hepatocellular carcinoma (HCC), as a life‐threatening disease, has become one of the most common malignancies and one of the leading causes of cancer death worldwide.^1^ Due to the lack of obvious symptoms in the early stages of HCC, more than 60% of patients with HCC are diagnosed in the advanced stages, leading to an extremely poor prognosis.^2^, ^3^ Therefore, the screening of a series of specific biomarkers is essential for the early diagnosis and prognostic evaluation of HCC. Interferon regulatory factors (IRFs) are a class of transcription factors that play a pivotal role in the regulation of innate and adaptive immune responses, as well as cellular processes linked to oncogenesis.^4^, ^5^ As a core member of the IRF family, IRF8 not only regulates the differentiation and maturation of a variety of immune cells, but also exerts antitumor activity by controlling various cell processes.^6^, ^7^, ^8^ An in‐depth exploration of the differences in expression of IRF8 and its correlation with immune cells in HCC is of great importance for predicting the clinical prognosis of HCC patients and screening appropriate diagnostic targets and intervention strategies.

In this report, we evaluated IRF8 transcription levels in multiple HCC studies based on the HCCDB database and found that the mRNA expression of IRF8 in 11 of 12 HCC cohorts was significantly lower in HCC tissues than in adjacent normal tissues (Figure 1A). Furthermore, we analyzed the correlation between IRF8 expression and clinic pathological characteristics of TCGA‐LIHC samples in the UALCAN database and found that the IRF8 transcription was negatively correlated with higher tumor stages and grades (Figure S1A–C). Furthermore, analysis of four HCC cohorts (“Chen Liver” data set, “Roessler Liver” data set, “Roessler Liver 2” data set, and “Wurmbach Liver” dataset) also revealed the downregulation of IRF8 expression in HCC tissues, which was further confirmed by meta‐analysis based on the ONCOMINE database (Figure S1D–H). In particular, we also found that the copy number of IRF8 was significantly lower in HCC tissues compared to normal tissues (Figure S1I–L).

To verify the expression level of IRF8, we explored IRF8 protein expression using 12 pairs of human HCC and matched adjacent tissues and found a lower expression of IRF8 in 8 cases of HCC than that in adjacent tissues (Figure 1B). In addition, we performed qPCR analyses in 20 paired HCC and adjacent tissues, and the results revealed that the mRNA level of IRF8 in HCC was also significantly lower than that in adjacent tissues (Figure 1C). Furthermore, we explored IRF8 expression in a cohort of 90 pairs of HCC and adjacent tissues with immunohistochemistry and observed lower levels of IRF8 in HCC compared with adjacent tissues (Figure 1D). In particularly, HCC patients with high IRF8 protein levels had longer overall survival compared with those with low IRF8 expression (Figure 1E). We further analyzed the relationship between IRF8 expression and the clinicopathological variables of HCC patients and found that IRF8 negatively correlated with serum AFP level and tumor size (Table S1). We also performed univariate and multivariate Cox regression analysis and found that the IRF8 expression was an independent prognostic factor for evaluating the overall survival of patients with HCC (Table S2).

**FIGURE 1:**
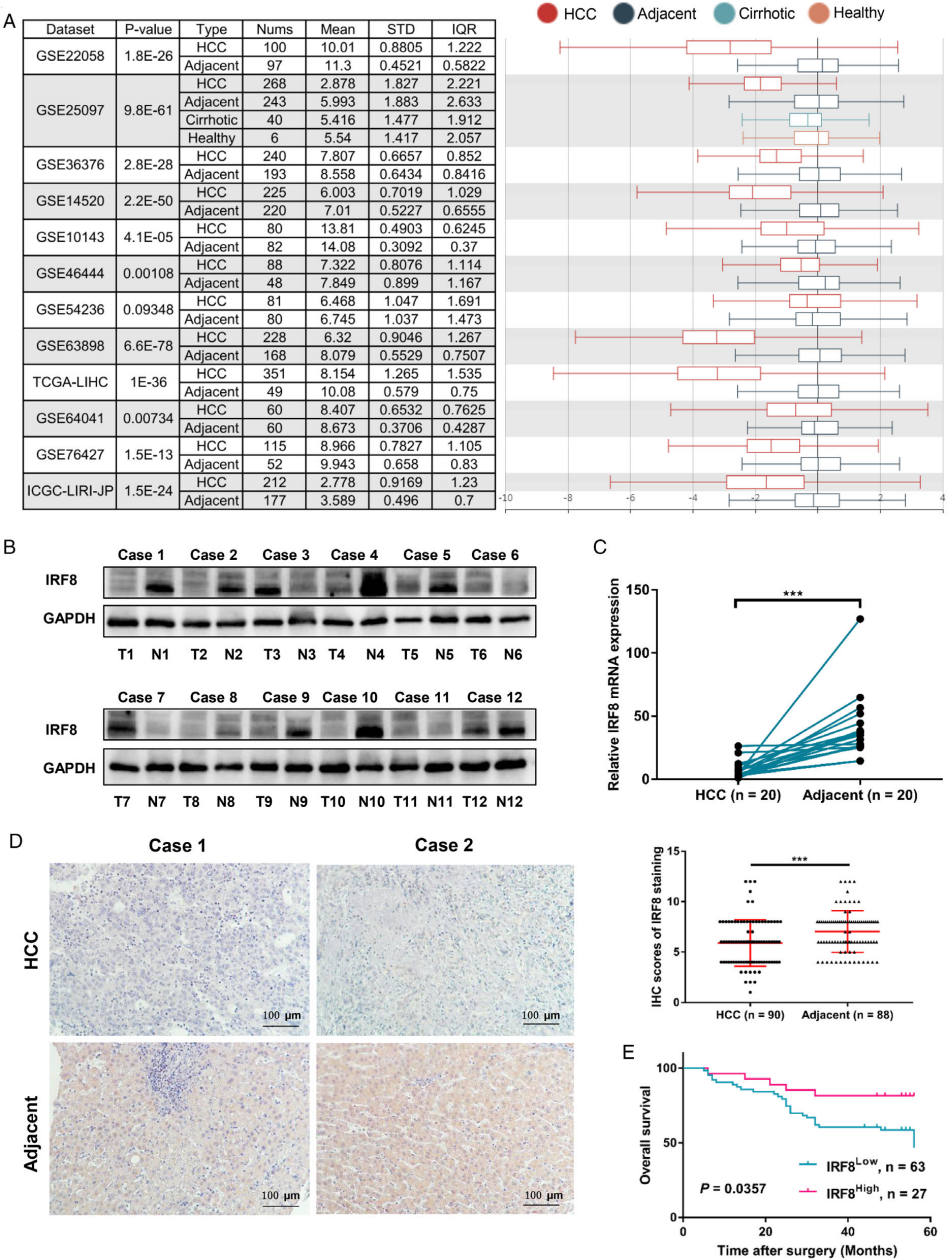
Downregulated expression and Kaplan–Meier survival analysis of IRF8 in HCC. (A) The expression of IRF8 in tumor and adjacent normal tissues was determined in multiple HCC cohorts based on the HCCDB database. (B) Western blot analysis of IRF8 protein expression in 12 pairs of human HCC and matched adjacent tissues. T, tumor; N, adjacent. (C) Quantitative PCR analysis of IRF8 mRNA expression in 20 pairs of HCC and matched adjacent tissues. (D) Immunohistochemical detection of IRF8 expression in HCC (*n* = 90) and adjacent tissues (*n* = 88). (E) Overall survival rates of 90 cases HCC patients with high or low IRF8 expression were evaluated by Kaplan–Meier analysis. (***p < 0.001)

To explore the biological meaning of IRF8 in HCC, the cBioPotral database was used to examine coexpressed genes with IRF8 in HCC. As shown in (Table S3), 129 genes with Spearman's correlation ≥ 0.5 were selected as coexpressed IRF8 genes. Then, the protein–protein interaction (PPI) network of these coexpression genes was built by Cytoscape and the MCODE plugin was employed to identify the modules in the PPI network (Figure S2A). The top 4 significant modules are displayed in (Figure S2B), and the seeds of four separate modules were CD53, ITGB2, HLA‐DPB1, and IL7R. GO and KEGG analysis of the IRF8 coexpressed genes indicated these were mainly involved in immune cell‐related biological processes and signaling pathways such as immune responses and inflammatory responses (Figure S2C and D).

To further clarify the expression of these four key genes in HCC, we evaluated their transcription levels in multiple HCC studies based on the HCCDB database. The results demonstrated that the levels of CD53, ITGB2, HLA‐DPB1, and IL7R mRNA expression were generally lower in HCC tissues than in adjacent normal tissues (Table S4). The downregulated expression of these four genes in HCC tissues was significant in 8, 8, 9, and 11 HCC cohorts, respectively (Table S4).

Tumor purity is an important factor that infers immune infiltration in tumor samples based on gene expression signatures.^9^ Given the key role of tumor‐infiltrating immune cells in tumor development, we investigated the relationship between the expression of the above five crucial genes and immune infiltration in LIHC using the Tumor IMmune Estimation Resource (TIMER). The results showed a significant negative correlation between tumor purity and IRF8, CD53, ITGB2, HLA‐DPB1, and IL7R expression (Figure S3A–E). Furthermore, the expression of the five key genes mentioned above were also significantly positively correlated with B cells, CD8+ T cells, CD4+ T cells, macrophages, neutrophils, and DCs (Figure S3A–E). Therefore, we speculate that the low expression of these five genes may play a key role in the antitumor immune responses of the HCC microenvironment.

To explore the relationship between IRF8 coexpressed genes and the survival of HCC patients, we performed Kaplan–Meier survival analysis based on TCGA database. The results showed that low expression of HLA‐DPB1, and IL7R significantly correlated with worse OS in HCC patients (Figure S4C and D). Furthermore, the low level of transcription of CD53, ITGB2, HLA‐DPB1, and IL7R significantly correlated with poor RFS, and PFS of patients with HCC, respectively (Figure S4A–D).

In summary, our study found that low expression of IRF8 and its four key coexpressed genes in HCC tissues were negatively correlated with tumor purity, and positively correlated with immune cell infiltration, and could be used to predict patient survival. This study is of great significance for the selection of new immune‐related targets, the assessment of prognostic response, and to guide clinicians in choosing appropriate treatments for patients with HCC.

## CONFLICT OF INTEREST

The authors declare no conflict of interest.

## AUTHOR CONTRIBUTIONS

Ding Wei, Huijie Bian, and Zhinan Chen conceived the study and revised the manuscript. Renyu Zhang, Yixiao Guo, and Zekun Liu performed most of the experiments, analyzed the data, and wrote the manuscript. Lingmin Kong, Can Li, Lin He, and Cong Zhang contributed to evaluate the data, prepare the figures, and review the literature. All authors read and approved the final manuscript.

## ETHICS STATEMENT

The study protocol was approved by the Institutional Ethics Review Board of the Fourth Military Medical University.

## Supporting information

Figure S1 IRF8 mRNA expression and copy number were downregulated in HCC
Figure S2 IRF8 coexpression networks and enrichment analysis in HCC
Figure S3 Correlation of IRF8 and the expression of the four seed genes with tumor purity and immune cell infiltration levels in LIHC
Figure S4 Kaplan–Meier survival analysis of IRF8 coexpressed genes in liver cancer samples
Table S1. Correlation of IRF8 expression and clinicopathological variables in patients with HCC
Table S2. Univariate and multivariate analyses of IRF8 expression in HCC
Table S3. IRF8 coexpression genes in HCC
Table S4. The expression levels of four crucial IRF8 coexpressed genes in HCCDB

## Data Availability

The data sets used and analyzed during the current study are available from the corresponding author upon reasonable request.
