# Supplementary material for: Identification of IRF8 as an immune infiltration‐related biomarker in hepatocellular carcinoma by bioinformatics analysis
Source: MedComm (2020). 2022 Jul 10;3(3):e149. doi: 10.1002/mco2.149 (PMC9271887; doi:10.1002/mco2.149)
Supplement: Supplementary file 1 — Figure S1 IRF8 mRNA expression and copy number were downregulated in HCC Figure S2 IRF8 coexpression networks and enrichment analysis in HCC Figure S3 Correlation of IRF8 and the expression of the four seed genes with tumor purity and immune cell infiltration levels in LIHC Figure S4 Kaplan–Meier survival analysis of IRF8 coexpressed genes in liver cancer samples Table S1. Correlation of IRF8 expression and clinicopathological variables in patients with HCC Table S2. Univariate and multivariate analyses of IRF8 expression in HCC Table S3. IRF8 coexpression genes in HCC Table S4. The expression levels of four crucial IRF8 coexpressed genes in HCCDB [file MCO2-3-e149-s001.docx]

Supplementary Materials for

Identification of IRF8 as an immune infiltration-related biomarker in hepatocellular carcinoma by bioinformatics analysis

Renyu Zhang**^†^**, Yixiao Guo**^†^**, Zekun Liu**^†^**, Lingmin Kong, Can Li, Lin He, Cong Zhang, Zhinan Chen*, Huijie Bian*, Ding Wei*

Correspondence to: Ding Wei: [weidcq@fmmu.edu.cn](mailto:weidcq@fmmu.edu.cn); Huijie Bian: hjbian@fmmu.edu.cn; Zhinan Chen: znchen@fmmu.edu.cn

**This PDF file includes:**

Materials and Methods

References

Figure S1 to S4

Table S1 to S4

**MATERIALS AND METHODS**

**HCCDB database analysis**

HCCDB is a HCC expression atlas including 15 public HCC gene expression datasets with a total of 3917 samples, including 13 microarray datasets from the Gene Expression Omnibus (GEO) database and 2 RNA-seq datasets, Liver Hepatocellular Carcinoma Project of The Cancer Genome Atlas (TCGA-LIHC) and Liver Cancer - RIKEN, JP Project from International Cancer Genome Consortium (ICGC LIRI-JP).[^1^](#_ENREF_1) The HCCDB database was used to analyze the differential expression of the IRF8, CD53, ITGB2, HLA-DPB1, and IL7R genes in tumor tissues and adjacent tissues based on multiple datasets.

**Western blotting analysis**

The 12 pairs of HCC and adjacent tissues used for western blotting analysis were obtained from the Xijing Hospital of Fourth Military Medical University. Proteins from lysed HCC and adjacent tissues were fractionated by SDS-PAGE and were transferred to PVDF membranes. The membranes were then blocked with 5% skimmed milk and incubated with primary antibodies including anti-IRF8 (83413T, Cell Signaling Technology) and anti-GAPDH (R1210-1, Huabio). After incubated with an HRP-conjugated secondary antibody, the protein was visualized using a Western-Light chemiluminescent detection system.

**Real-Time qPCR**

The 20 pairs of HCC and adjacent tissues used for qPCR analysis were obtained from the Xijing Hospital of Fourth Military Medical University. RNA was extracted using a Total RNA Kit II (Omega, Riverside, USA) and reverse-transcribed into complementary DNA by PrimeScript RT reagent kit (TaKaRa, Otsu, Japan). Single-stranded complementary DNA was amplified by quantitative RT-PCR using a SYBR Premix ExTaq kit (TaKaRa) on the Stratagene Mx3005P Real-Time PCR System. The primer sequences were as follows: IRF8-forward (AGGTCTTCGACACCAGCCAGTT), IRF8-reverse (GCACGAGAATGAGTTTGGAGCG); β-actin-forward (CACCATTGGCAATGAGCGGTTC), β-actin-reverse (AGGTCTTTGCGGATGTCCACGT).

**ONCOMINE database analysis**

ONCOMINE ([www.oncomine.org](http://www.oncomine.org)) is a publicly accessible online cancer microarray database that can comprehensively analyze gene expression differences. The mRNA levels and copy number variation of IRF8 in four HCC cohorts (‘Chen Liver’ dataset, ‘Roessler Liver’ dataset, ‘Roessler Liver 2' dataset and ‘Wurmbach Liver’ dataset) were determined by analysis in the ONCOMINE database.

**UALCAN database analysis**

UALCAN (<http://ualcan.path.uab.edu>) is a comprehensive and interactive web resource for analyzing cancer OMICS data (TCGA, MET500, and CPTAC).[^2^](#_ENREF_2) UALCAN was used to analyze the relative expression level of IRF8 in TCGA database across normal and tumor samples, as well as in various subgroups based on individual tumor stage and grade.

**Immunohistochemistry**

A tissue microarray (HLivH180Su16) containing 90 pairs of HCC and corresponding adjacent tissues were purchased from Shanghai Outdo Biotech Co., Ltd. Paraffin-embedded sections were deparaffinized, rehydrated, followed by antigen retrieval with citrate buffer (pH 6.0). Sections were treated with methanol containing 3% hydrogen peroxide for 10 min, washed with PBS, and blocked with goat serum for 30 min. Then, sections were incubated with anti-IRF8 (83413T, Cell Signaling Technology) diluted 1:250, at 4ºC overnight. Immunoperoxidase staining was performed using a streptavidin-peroxidase kit and 3,3’-diaminobenzidine (Zhongshan Jinqiao Co., Beijing, China). Further, the nuclei was counterstained with hematoxylin. The staining intensity was graded as follows: negative = 0; weak = 1; moderate = 2; strong = 3. The staining percentage was graded as follows: <5% = 0; 5%-25% = 1; 26%-50% = 2; 51%-75% = 3; >75% = 4. The final staining score, which was categorized as low (0-7) or high (8-12), was determined by multiplying the score for intensity and percentage.

**Protein-protein interaction network analysis**

The STRING database (<https://string-db.org/>) was used to calculate the protein-protein interaction (PPI) network of IRF8 co-expressed genes. The PPI network was further visualized using Cytoscape software (version3.4.0). MCODE was a Cytoscape plugin for constructing the protein module complex with a degree cut-off = 2, node score cut-off = 0.2, k-core = 2, and max. depth = 100.

**Enrichment analysis**

DAVID (<https://david.ncifcrf.gov/>) is an online set of functional annotation tools that reveal the biological activities of a list of genes.[^3^](#_ENREF_3) Based on IRF8 and its co-expressed genes, DAVID v6.8 was used to perform GO enrichment to explore biological processes, cellular components, and molecular function, and was used to perform KEGG analyses to identify biological pathways. *P* < 0.05 denoted statistical significance.

**TIMER database analysis**

TIMER (<https://cistrome.shinyapps.io/timer/>) is a comprehensive resource for systematic analysis of tumor-infiltrating immune cells in various types of cancer.[^4^](#_ENREF_4) TIMER was used to analyze the correlation between IRF8, CD53, ITGB2, HLA-DPB1, and IL7R expression with tumor purity or immune infiltration cells in HCC.

**Kaplan-Meier plotter analysis**

The Kaplan-Meier plotter (http://kmplot.com/analysis/) is capable of assessing the correlation between the expression of 30k genes (mRNA, miRNA, and protein) and survival in 21 cancer types including breast, ovarian, lung, gastric, and liver cancer.[^5^](#_ENREF_5) The correlation between CD53, ITGB2, HLA-DPB1, or IL7R expression and overall survival (OS), relapse-free survival (RFS), or progression-free survival (PFS) in liver cancer was analyzed by the Kaplan-Meier plotter.

**Statistical analysis**

Data were analyzed using GraphPad Prism 7.0. Survival rate was calculated using Kaplan–Meier analysis and log-rank test. The correlation between the IRF8 expression and clinicopathological variables was computed using χ2 test. The statistical analysis was performed as appropriate by Student's *t* test. *P* < 0.05 was considered statistically significant.

**REFERENCES**

1. Lian Q, Wang S, Zhang G, et al. HCCDB: A database of hepatocellular carcinoma expression atlas. *Genomics Proteomics Bioinformatics*. 2018;16(4):269-275.

2. Chandrashekar DS, Bashel B, Balasubramanya SAH, et al. UALCAN: A portal for facilitating tumor subgroup gene expression and survival analyses. *Neoplasia*. 2017;19(8):649-658.

3. Dennis G, Jr., Sherman BT, Hosack DA, et al. DAVID: database for annotation, visualization, and integrated discovery. *Genome Biol.* 2003;4(5):P3.

4. Li T, Fan J, Wang B, et al. TIMER: a web server for comprehensive analysis of tumor-infiltrating immune cells. *Cancer Res.* 2017;77(21):e108-e110.

5. Lanczky A, Gyorffy B. Web-based survival analysis tool tailored for medical research (KMplot): development and implementation. *J Med Internet Res.* 2021;23(7):e27633.


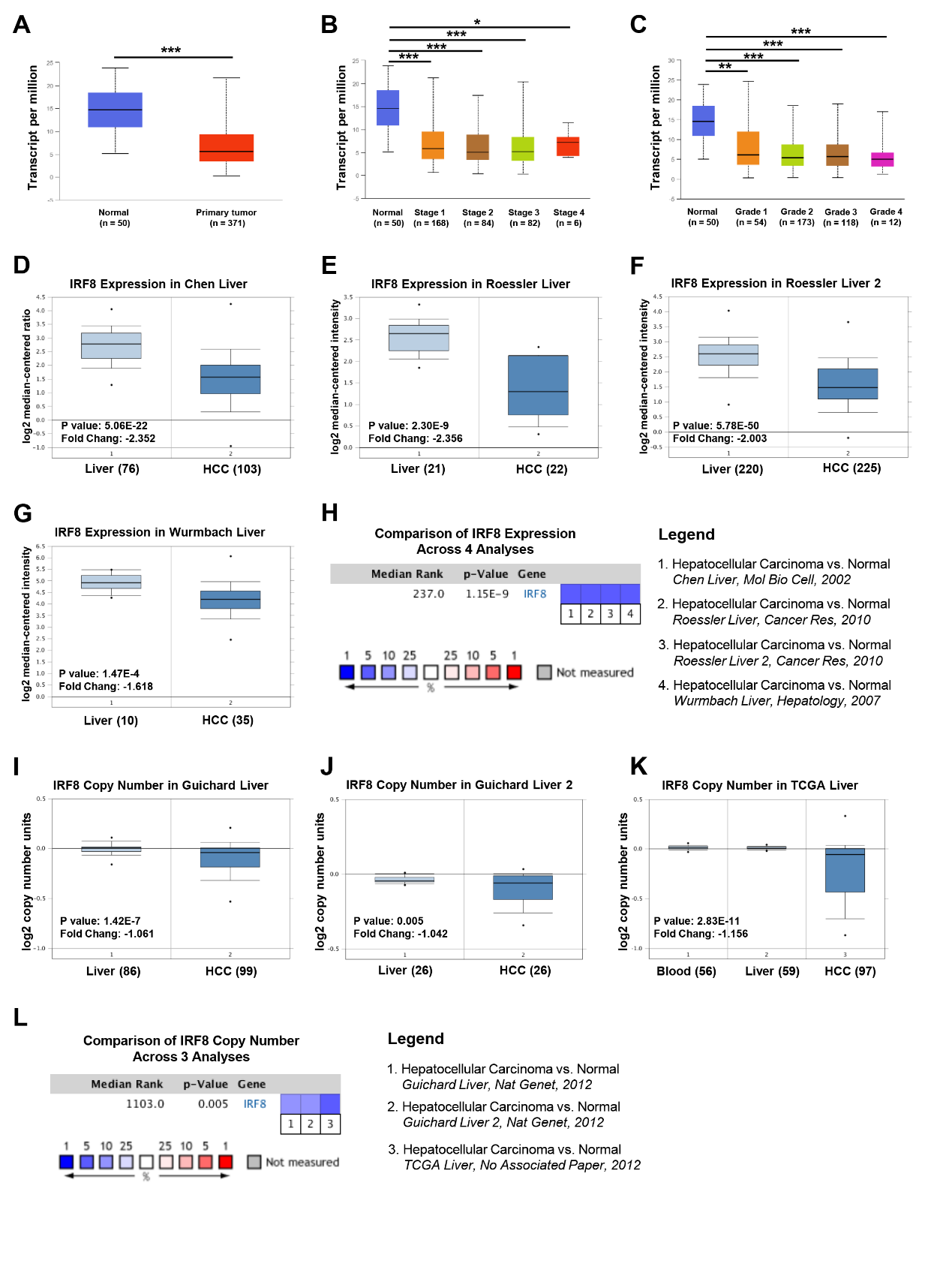
**Figure S1** IRF8 mRNA expression and copy number were down-regulated in HCC. **(A-C)** IRF8 transcription in subgroups of patients with HCC, stratified according to tumor stage and grade (UALCAN database). **(D-G)** Comparison of IRF8 mRNA expression in normal and HCC tissues (ONCOMINE database). **(H)** Meta-analysis of down-regulated expression of IRF8 in four HCC cohorts (ONCOMINE database). **(I-K)** Comparison of IRF8 copy number in normal and HCC tissues (ONCOMINE database). **(L)** Meta-analysis of the down-regulated copy number of IRF8 in three HCC cohorts (ONCOMINE database).


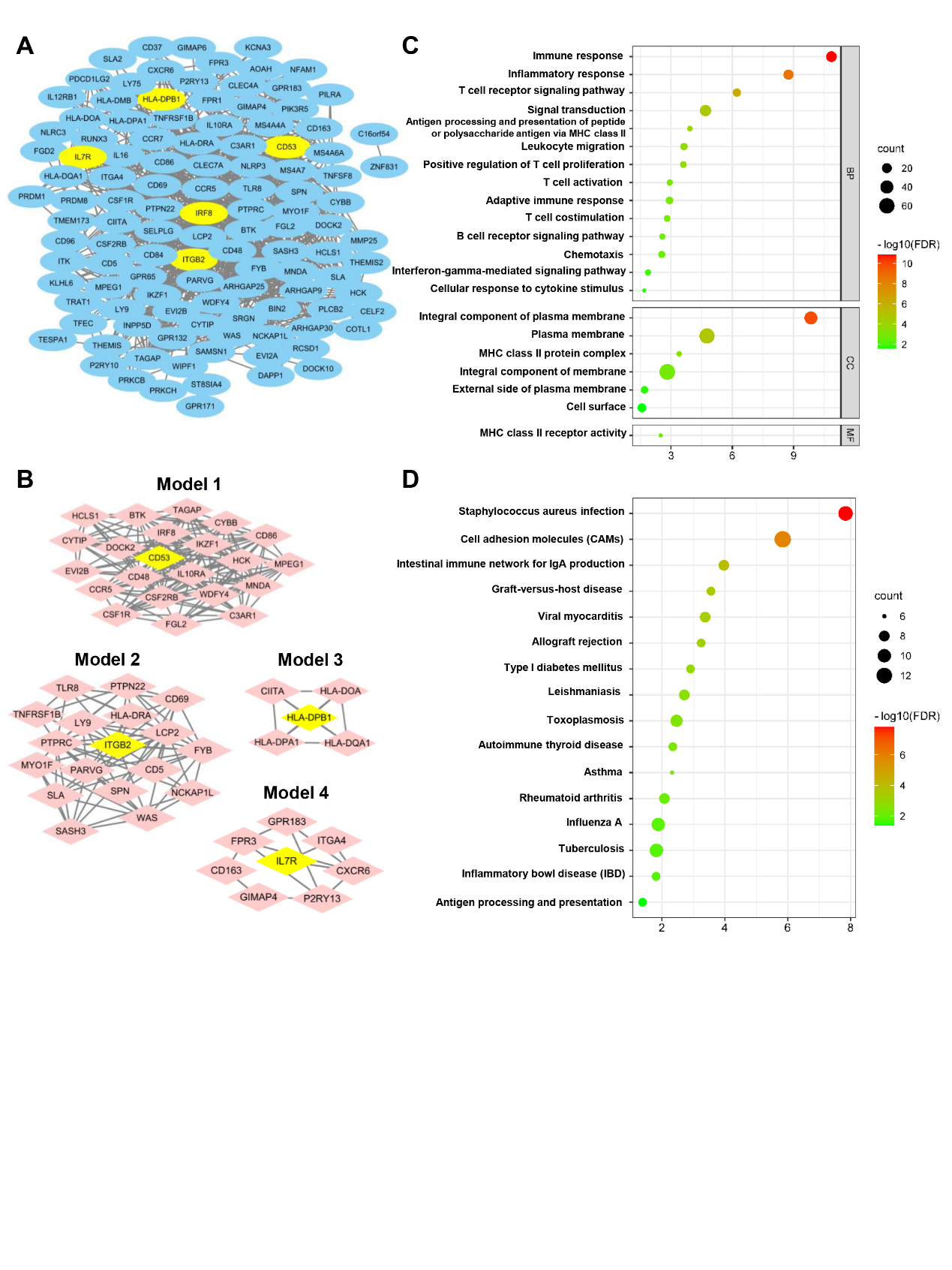
**Figure S2** IRF8 co-expression networks and enrichment analysis in HCC. **(A)** Global IRF8 highly correlated genes with Spearman's correlation ≥ 0.5 in LIHC (cBioPotral database). The PPI network of these co-expressed genes was visualized by Cytoscape. **(B)** The MCODE plugin was used to identify the modules in the PPI network. Yellow indicates seed genes. **(C-D)** GO and KEGG enrichments were used to analyze IRF8 and co-expressed genes. *P* < 0.01, FDR < 0.05.


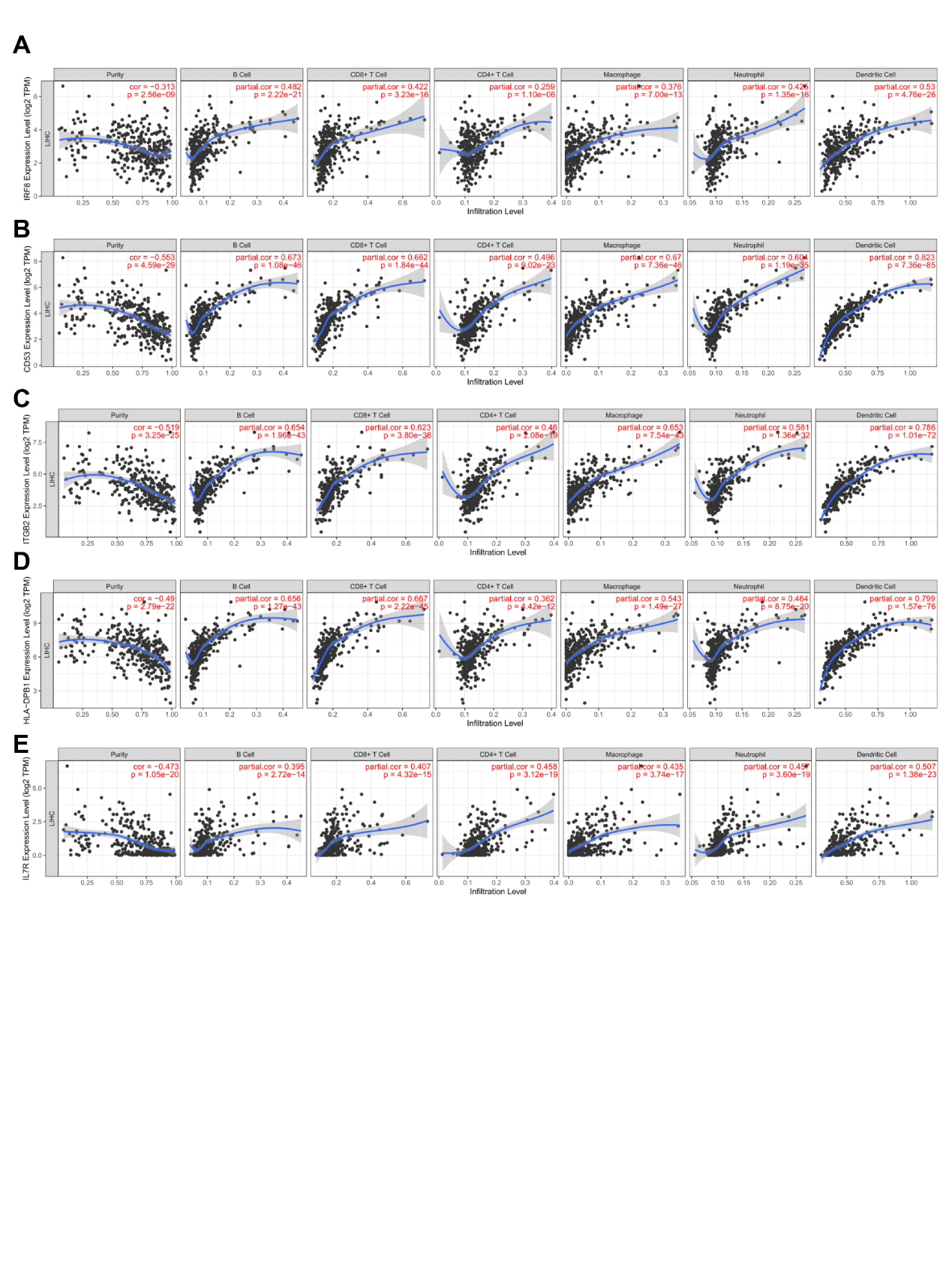
**Figure S3** Correlation of IRF8 and the expression of the four seed genes with tumor purity and immune cell infiltration levels in LIHC. **(A-E)** IRF8, CD53, ITGB2, HLA-DPB1 and IL7R were all significantly negatively related to tumor purity and had significant positive correlations with infiltrating levels of B cells, CD8+ T cells, CD4+ T cells, macrophages, neutrophils, and dendritic cells in LIHC.


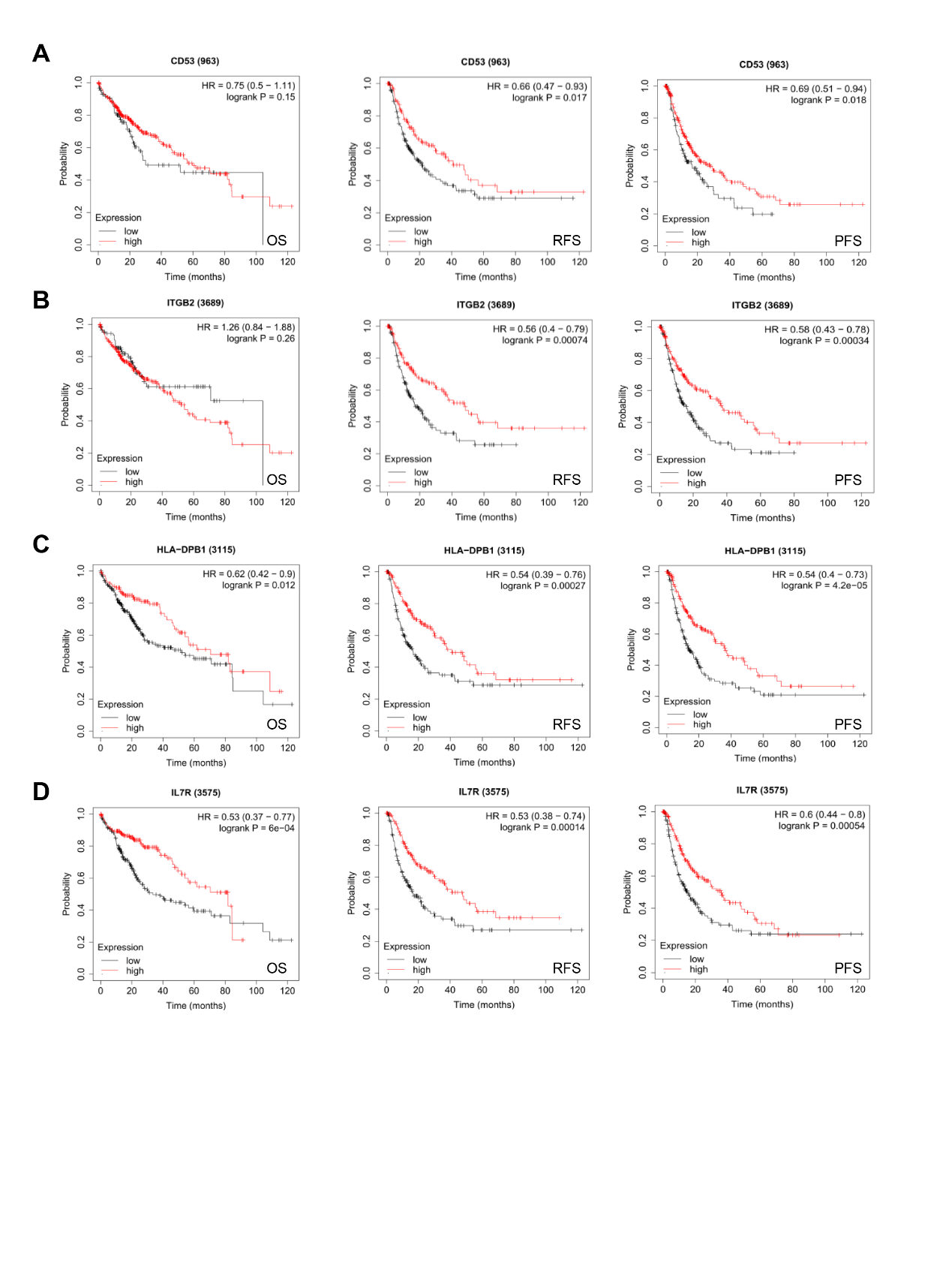
**Figure S4** Kaplan-Meier survival analysis of IRF8 co-expressed genes in liver cancer samples. The prognostic OS, RFS, and PFS analyses of CD53 **(A)**, ITGB2 **(B)**, HLA-DPB1 **(C)**, and IL7R **(D)** were performed in 364, 316, and 370 live cancer samples, respectively.

Table S1. Correlation of IRF8 expression and clinicopathologic variables in patients with HCC.

| Clinicopathological variables | IRF8 expression | | *P* value |
| --- | --- | --- | --- |
|  | High (n = 27) | Low (n = 63) | |
| Age |  |  |  |
| <50 years | 11 | 26 |  |
| ≥50 years | 16 | 37 | 0.963 |
| Gender |  |  |  |
| Male | 25 | 55 |  |
| Female | 2 | 8 | 0.714 |
| Grade |  |  |  |
| I-II | 17 | 26 |  |
| III | 10 | 37 | 0.059 |
| Serum AFP | |  |  |
| NA | 0 | 1 |  |
| <20 (ng/mL) | 16 | 20 |  |
| ≥20 (ng/mL) | 11 | 42 | **0.017** |
| HBsAg |  |  |  |
| NA | 0 | 1 |  |
| Positive | 22 | 48 |  |
| Negative | 5 | 14 | 0.667 |
| TNM stage | |  |  |
| I | 21 | 42 |  |
| II-III | 6 | 21 | 0.292 |
| Tumor number | |  |  |
| Single | 24 | 55 |  |
| Multiple | 3 | 8 | 1.000 |
| Tumor size | |  |  |
| <4.5 cm | 19 | 29 |  |
| ≥4.5 cm | 8 | 34 | **0.034** |

AFP, alpha-fetoprotein; HBsAg, hepatitis B virus surface antigen; TNM, tumor-node metastasis.

Table S2. Univariate and multivariate analyses of IRF8 expression in HCC.

| Clinicopathological characteristics | Univariate analyses (OS, n = 90) | | | Multivariate analyses (OS, n = 90) | | |
| --- | --- | --- | --- | --- | --- | --- |
|  | HR | 95% CI | *P* value | HR | 95% CI | *P* value |
| Age | 1.167 | 0.570-2.388 | 0.673 |  |  |  |
| Gender | 1.942 | 0.464-8.128 | 0.364 |  |  |  |
| Grade | 3.050 | 1.393-6.681 | **0.005** | 2.153 | 0.953-4.866 | 0.065 |
| Serum AFP | 1.336 | 0.639-2.794 | 0.441 |  |  |  |
| HBsAg | 0.961 | 0.410-2.252 | 0.927 |  |  |  |
| TNM stage | 2.285 | 1.139-4.581 | **0.020** | 1.454 | 0.711-2.972 | 0.305 |
| Tumor number | 1.833 | 0.751-4.474 | 0.183 |  |  |  |
| Tumor size | 2.280 | 1.111-4.682 | **0.025** | 1.458 | 0.684-3.107 | 0.329 |
| IRF8 expression | 0.673 | 0.557-0.812 | **0.000** | 0.737 | 0.603-0.900 | **0.003** |

OS, overall survival; HR, hazard ratio; CI, confidence interval.

Table S3. IRF8 co-expression genes in HCC.

| Correlated Gene | Spearman's Correlation | p-Value | q-Value |
| --- | --- | --- | --- |
| TAGAP | 0.588 | 4.56E-36 | 9.16E-32 |
| THEMIS2 | 0.581 | 4.10E-35 | 4.12E-31 |
| CLEC7A | 0.579 | 8.96E-35 | 6.00E-31 |
| FGL2 | 0.577 | 1.67E-34 | 8.41E-31 |
| DOCK2 | 0.573 | 5.47E-34 | 2.09E-30 |
| HLA-DOA | 0.573 | 6.23E-34 | 2.09E-30 |
| AOAH | 0.571 | 1.15E-33 | 3.29E-30 |
| ARHGAP30 | 0.567 | 4.56E-33 | 1.15E-29 |
| TLR8 | 0.565 | 8.11E-33 | 1.81E-29 |
| NFAM1 | 0.564 | 1.07E-32 | 2.16E-29 |
| CSF2RB | 0.563 | 1.48E-32 | 2.55E-29 |
| SLA | 0.563 | 1.53E-32 | 2.55E-29 |
| RUNX3 | 0.562 | 1.88E-32 | 2.71E-29 |
| CYTIP | 0.562 | 1.89E-32 | 2.71E-29 |
| FPR3 | 0.56 | 3.17E-32 | 3.99E-29 |
| PTPRC | 0.56 | 3.17E-32 | 3.99E-29 |
| KLHL6 | 0.56 | 3.65E-32 | 4.08E-29 |
| SRGN | 0.56 | 3.78E-32 | 4.08E-29 |
| HLA-DPA1 | 0.56 | 3.86E-32 | 4.08E-29 |
| CELF2 | 0.558 | 5.92E-32 | 5.81E-29 |
| SAMSN1 | 0.558 | 6.08E-32 | 5.81E-29 |
| ITK | 0.557 | 9.61E-32 | 8.78E-29 |
| HCLS1 | 0.554 | 2.06E-31 | 1.80E-28 |
| GPR171 | 0.554 | 2.35E-31 | 1.97E-28 |
| KCNA3 | 0.551 | 6.01E-31 | 4.83E-28 |
| MS4A6A | 0.549 | 1.01E-30 | 7.78E-28 |
| EVI2B | 0.548 | 1.21E-30 | 9.00E-28 |
| IKZF1 | 0.548 | 1.39E-30 | 9.98E-28 |
| TNFRSF1B | 0.545 | 3.15E-30 | 2.18E-27 |
| IL7R | 0.545 | 3.43E-30 | 2.29E-27 |
| CD53 | 0.544 | 3.72E-30 | 2.41E-27 |
| WAS | 0.544 | 4.18E-30 | 2.63E-27 |
| DOCK10 | 0.544 | 4.70E-30 | 2.86E-27 |
| MPEG1 | 0.543 | 5.39E-30 | 3.18E-27 |
| SCIMP | 0.543 | 5.84E-30 | 3.35E-27 |
| PTPN22 | 0.543 | 6.14E-30 | 3.43E-27 |
| HLA-DMB | 0.542 | 7.79E-30 | 4.23E-27 |
| FPR1 | 0.54 | 1.16E-29 | 6.15E-27 |
| C16ORF54 | 0.54 | 1.29E-29 | 6.67E-27 |
| TRAT1 | 0.539 | 1.67E-29 | 8.41E-27 |
| INPP5D | 0.539 | 1.93E-29 | 9.46E-27 |
| EVI2A | 0.538 | 2.15E-29 | 1.01E-26 |
| FYB1 | 0.538 | 2.17E-29 | 1.01E-26 |
| LCP2 | 0.538 | 2.39E-29 | 1.09E-26 |
| GPR65 | 0.538 | 2.54E-29 | 1.13E-26 |
| SPN | 0.537 | 2.95E-29 | 1.29E-26 |
| MYO1F | 0.536 | 3.47E-29 | 1.48E-26 |
| PIK3R5 | 0.535 | 5.28E-29 | 2.21E-26 |
| PRKCB | 0.533 | 8.56E-29 | 3.51E-26 |
| PTGER2 | 0.532 | 1.18E-28 | 4.75E-26 |
| HLA-DRA | 0.532 | 1.33E-28 | 5.24E-26 |
| GPR183 | 0.531 | 1.55E-28 | 5.97E-26 |
| CIITA | 0.531 | 1.78E-28 | 6.75E-26 |
| PLCB2 | 0.53 | 1.89E-28 | 7.03E-26 |
| PRKCH | 0.53 | 1.95E-28 | 7.13E-26 |
| FCMR | 0.529 | 2.42E-28 | 8.70E-26 |
| HACD4 | 0.529 | 2.48E-28 | 8.73E-26 |
| ZNF831 | 0.529 | 2.92E-28 | 1.01E-25 |
| SLA2 | 0.529 | 3.06E-28 | 1.04E-25 |
| RCSD1 | 0.528 | 3.59E-28 | 1.20E-25 |
| CYBB | 0.528 | 4.17E-28 | 1.37E-25 |
| RASSF2 | 0.527 | 4.38E-28 | 1.42E-25 |
| ITGA4 | 0.526 | 6.93E-28 | 2.21E-25 |
| IL10RA | 0.526 | 7.10E-28 | 2.23E-25 |
| NCKAP1L | 0.525 | 7.28E-28 | 2.25E-25 |
| HCK | 0.525 | 7.51E-28 | 2.29E-25 |
| CD69 | 0.525 | 7.70E-28 | 2.31E-25 |
| PRDM1 | 0.525 | 8.39E-28 | 2.48E-25 |
| KCTD12 | 0.525 | 9.16E-28 | 2.67E-25 |
| CCR5 | 0.524 | 1.18E-27 | 3.39E-25 |
| DAPP1 | 0.524 | 1.22E-27 | 3.42E-25 |
| BIN2 | 0.524 | 1.23E-27 | 3.42E-25 |
| NLRC3 | 0.523 | 1.55E-27 | 4.27E-25 |
| CD5 | 0.522 | 2.05E-27 | 5.54E-25 |
| P2RY13 | 0.522 | 2.07E-27 | 5.54E-25 |
| SASH3 | 0.522 | 2.11E-27 | 5.56E-25 |
| IL16 | 0.521 | 2.29E-27 | 5.96E-25 |
| MNDA | 0.52 | 2.78E-27 | 7.16E-25 |
| HLA-DQA1 | 0.52 | 3.07E-27 | 7.82E-25 |
| MMP25 | 0.52 | 3.41E-27 | 8.57E-25 |
| FGD2 | 0.519 | 4.17E-27 | 1.03E-24 |
| GPR132 | 0.519 | 4.31E-27 | 1.06E-24 |
| CLEC4A | 0.518 | 4.85E-27 | 1.17E-24 |
| COTL1 | 0.518 | 5.29E-27 | 1.26E-24 |
| TFEC | 0.517 | 7.10E-27 | 1.68E-24 |
| HLA-DPB1 | 0.516 | 8.64E-27 | 2.02E-24 |
| GIMAP4 | 0.515 | 1.09E-26 | 2.51E-24 |
| CMKLR1 | 0.515 | 1.13E-26 | 2.57E-24 |
| THEMIS | 0.515 | 1.15E-26 | 2.60E-24 |
| GIMAP6 | 0.515 | 1.22E-26 | 2.73E-24 |
| NLRP3 | 0.514 | 1.54E-26 | 3.41E-24 |
| ITGB2 | 0.513 | 1.79E-26 | 3.90E-24 |
| TMEM173 | 0.513 | 1.96E-26 | 4.23E-24 |
| EMB | 0.513 | 2.08E-26 | 4.46E-24 |
| PDCD1LG2 | 0.512 | 2.29E-26 | 4.84E-24 |
| CD84 | 0.512 | 2.34E-26 | 4.89E-24 |
| C3AR1 | 0.511 | 3.69E-26 | 7.64E-24 |
| CCR7 | 0.51 | 4.01E-26 | 8.23E-24 |
| WIPF1 | 0.51 | 4.27E-26 | 8.66E-24 |
| SLC8A1 | 0.51 | 4.88E-26 | 9.80E-24 |
| SELPLG | 0.509 | 5.29E-26 | 1.05E-23 |
| PARVG | 0.509 | 5.60E-26 | 1.10E-23 |
| SIDT1 | 0.508 | 6.53E-26 | 1.27E-23 |
| CD48 | 0.508 | 6.72E-26 | 1.30E-23 |
| PRDM8 | 0.508 | 7.26E-26 | 1.39E-23 |
| CSF1R | 0.508 | 7.57E-26 | 1.43E-23 |
| LY75 | 0.507 | 9.80E-26 | 1.84E-23 |
| SIRPB2 | 0.507 | 1.02E-25 | 1.90E-23 |
| GVINP1 | 0.506 | 1.16E-25 | 2.13E-23 |
| MS4A7 | 0.505 | 1.45E-25 | 2.65E-23 |
| CD86 | 0.505 | 1.46E-25 | 2.65E-23 |
| PPP1R16B | 0.504 | 1.93E-25 | 3.46E-23 |
| WDFY4 | 0.504 | 2.01E-25 | 3.57E-23 |
| TESPA1 | 0.504 | 2.08E-25 | 3.67E-23 |
| TNFSF8 | 0.503 | 2.28E-25 | 3.98E-23 |
| PILRA | 0.503 | 2.33E-25 | 4.03E-23 |
| LY9 | 0.503 | 2.74E-25 | 4.70E-23 |
| CD37 | 0.503 | 2.90E-25 | 4.93E-23 |
| P2RY10 | 0.502 | 3.11E-25 | 5.25E-23 |
| BTK | 0.502 | 3.23E-25 | 5.41E-23 |
| MS4A4A | 0.502 | 3.34E-25 | 5.51E-23 |
| ARHGAP9 | 0.502 | 3.35E-25 | 5.51E-23 |
| CXCR6 | 0.502 | 3.51E-25 | 5.73E-23 |
| ST8SIA4 | 0.501 | 4.04E-25 | 6.54E-23 |
| CD163 | 0.501 | 4.11E-25 | 6.61E-23 |
| IL12RB1 | 0.501 | 4.17E-25 | 6.65E-23 |
| CD96 | 0.5 | 4.97E-25 | 7.85E-23 |
| ARHGAP25 | 0.5 | 5.34E-25 | 8.38E-23 |
| CXORF21 | 0.5 | 5.64E-25 | 8.79E-23 |

Table S4. The expression levels of four crucial IRF8 co-expressed genes in HCCDB.

| Dataset | Type | Nums | CD53 | | | |  | ITGB2 | | | |  | HLA-DPB1 | | | |  | IL7R | | | |
| --- | --- | --- | --- | --- | --- | --- | --- | --- | --- | --- | --- | --- | --- | --- | --- | --- | --- | --- | --- | --- | --- |
|  |  |  | P-value | Mean | STD | IQR |  | P-value | Mean | STD | IQR |  | P-value | Mean | STD | IQR |  | P-value | Mean | STD | IQR |
| GSE22058 | HCC | 100 | 4.76E-12 | 10.79 | 0.9886 | 1.417 |  | 3.33E-06 | 10.52 | 0.99 | 1.038 |  | 5.75E-12 | 7.657 | 1.241 | 1.73 |  | 1.42E-14 | 7.392 | 1.726 | 2.501 |
|  | Adjacent | 97 |  | 11.6 | 0.4068 | 0.4986 |  |  | 11.05 | 0.4867 | 0.7156 |  |  | 8.706 | 0.6432 | 0.8408 |  |  | 9.013 | 0.7138 | 0.9948 |
| GSE25097 | HCC | 268 | 2.35E-15 | 4.087 | 3.159 | 3.618 |  | 0.04311 | 3.382 | 3.655 | 2.582 |  | 1.76E-15 | 0.4665 | 0.4815 | 0.4292 |  | 4.45E-21 | 1.008 | 1.266 | 1.109 |
|  | Adjacent | 243 |  | 5.996 | 2.028 | 2.591 |  |  | 3.885 | 1.682 | 2.064 |  |  | 0.7841 | 0.3907 | 0.4885 |  |  | 2.063 | 1.156 | 1.346 |
|  | Cirrhotic | 40 |  | 10.2 | 2.914 | 4.166 |  |  | 6.815 | 3.105 | 5.032 |  |  | 1.317 | 0.6742 | 0.7123 |  |  | 2.696 | 1.073 | 1.579 |
|  | Healthy | 6 |  | 3.723 | 3.088 | 1.867 |  |  | 2.665 | 1.64 | 1.574 |  |  | 0.2018 | 0.1194 | 0.1058 |  |  | 1.122 | 0.8345 | 0.7159 |
| GSE36376 | HCC | 240 | 0.01637 | 6.71 | 0.3779 | 0.5197 |  | 0.06566 | 9.694 | 1.01 | 1.425 |  | 0.913 | 6.758 | 0.5793 | 0.8248 |  | 0.6729 | 7 | 0.6833 | 0.8004 |
|  | Adjacent | 193 |  | 6.634 | 0.278 | 0.3909 |  |  | 9.549 | 0.6119 | 0.8201 |  |  | 6.753 | 0.4096 | 0.5558 |  |  | 7.023 | 0.4346 | 0.4994 |
| GSE14520 | HCC | 225 | 0.001012 | 6.474 | 1.053 | 1.508 |  | 0.001178 | 5.831 | 0.9993 | 1.406 |  | 3.24E-10 | 8.613 | 1.155 | 1.566 |  | 2.26E-23 | 5.914 | 0.8225 | 0.976 |
|  | Adjacent | 220 |  | 6.777 | 0.8695 | 1.215 |  |  | 6.096 | 0.6931 | 0.9657 |  |  | 9.203 | 0.7276 | 0.9675 |  |  | 6.68 | 0.7036 | 0.8377 |
| GSE10143 | HCC | 80 | 0.7434 | 12.24 | 0.6712 | 0.9582 |  | 0.003063 | 13.12 | 0.7263 | 0.7398 |  | 0.0251 | 12.67 | 0.6711 | 0.7002 |  | 0.000168 | 11.38 | 0.9585 | 1.373 |
|  | Adjacent | 82 |  | 12.28 | 0.7428 | 1.117 |  |  | 13.42 | 0.4997 | 0.5207 |  |  | 12.91 | 0.7021 | 1.026 |  |  | 11.98 | 1.044 | 1.49 |
| GSE46444 | HCC | 88 | 0.01422 | 7.086 | 0.9604 | 0.9926 |  | 0.001222 | 9.344 | 0.7348 | 1.018 |  | 0.02172 | 6.111 | 0.9554 | 1.474 |  | 0.01535 | 7.561 | 0.913 | 0.9794 |
|  | Adjacent | 48 |  | 7.468 | 0.792 | 0.9651 |  |  | 9.772 | 0.7075 | 0.7949 |  |  | 6.602 | 1.269 | 2.279 |  |  | 7.992 | 1.003 | 1.033 |
| GSE54236 | HCC | 81 | 0.007547 | 9.61 | 1.057 | 1.318 |  | 0.178 | 8.444 | 0.8172 | 1.094 |  | 0.2234 | 14.09 | 1.059 | 1.255 |  | 2.21E-05 | 8.328 | 1.959 | 2.518 |
|  | Adjacent | 80 |  | 10.01 | 0.7793 | 1.046 |  |  | 8.601 | 0.6537 | 0.6941 |  |  | 14.3 | 1.118 | 1.08 |  |  | 9.574 | 1.64 | 1.926 |
| GSE63898 | HCC | 228 | 2.17E-26 | 5.56 | 1.031 | 1.362 |  | 2.09E-12 | 6.368 | 1.121 | 1.505 |  | 3.64E-21 | 7.421 | 1.218 | 1.762 |  | 7.79E-28 | 4.736 | 0.9771 | 0.8627 |
|  | Adjacent | 168 |  | 6.851 | 1.14 | 1.802 |  |  | 7.113 | 0.9202 | 1.456 |  |  | 8.618 | 1.137 | 1.793 |  |  | 6.204 | 1.322 | 2.301 |
| TCGA-LIHC | HCC | 351 | 0.0000131 | 8.177 | 1.349 | 1.845 |  | 0.0000269 | 9.656 | 1.305 | 1.745 |  | 1.11E-05 | 10.84 | 1.406 | 1.93 |  | 2.32E-09 | 3.747 | 2.307 | 3.435 |
|  | Adjacent | 49 |  | 8.962 | 1.06 | 1.08 |  |  | 10.29 | 0.8651 | 1.12 |  |  | 11.48 | 0.8204 | 0.78 |  |  | 5.66 | 1.767 | 2.24 |
| GSE64041 | HCC | 60 | 0.4058 | 9.673 | 0.7928 | 1.108 |  | 0.5317 | 8.47 | 0.6923 | 0.8483 |  | 0.4677 | 11.47 | 0.6188 | 0.7825 |  | 0.001242 | 7.142 | 1.46 | 2.335 |
|  | Adjacent | 60 |  | 9.778 | 0.5575 | 0.768 |  |  | 8.407 | 0.3491 | 0.425 |  |  | 11.54 | 0.5143 | 0.5755 |  |  | 7.937 | 1.153 | 1.718 |
| GSE76427 | HCC | 115 | 0.1243 | 7.561 | 0.6045 | 0.6975 |  | 0.01302 | 10.67 | 0.9742 | 1.185 |  | 0.03794 | 7.575 | 0.5991 | 0.82 |  | 0.002558 | 7.654 | 0.8243 | 1.133 |
|  | Adjacent | 52 |  | 7.721 | 0.624 | 0.6875 |  |  | 11.07 | 0.928 | 1.069 |  |  | 7.765 | 0.5102 | 0.6825 |  |  | 8.087 | 0.8421 | 1.205 |
| ICGC-LIRI-JP | HCC | 212 | 0.005393 | 3.817 | 1.331 | 1.865 |  | 0.9558 | 3.495 | 1.329 | 1.912 |  | 0.0148 | 4.49 | 1.382 | 2.067 |  | 4.38E-10 | 1.244 | 1.132 | 1.513 |
|  | Adjacent | 177 |  | 4.136 | 0.9057 | 1.36 |  |  | 3.501 | 0.8257 | 1.08 |  |  | 4.781 | 0.9547 | 1.26 |  |  | 1.956 | 1.057 | 1.44 |
